# Circ_0001174 facilitates osteosarcoma cell proliferation, migration, and invasion by targeting the miR-186-5p/MACC1 axis

**DOI:** 10.1186/s13018-022-03059-8

**Published:** 2022-03-12

**Authors:** Feifei Lin, Xiaonan Wang, Xin Zhao, Ming Ren, Qingyu Wang, Jincheng Wang

**Affiliations:** 1 Department of Orthopedics, The Second Hospital of Jilin University, Ziqiang Street 218, Changchun, 130041 Jilin China; 2 Research Centre, The Second Hospital of Jilin University, Ziqiang Street 218, Changchun, 130041 Jilin China

**Keywords:** Osteosarcoma, circRNA, miR-186-5p, MACC1, Differentially expressed genes

## Abstract

**Background:**

Studies of aberrantly expressed circular RNAs (circRNAs) can provide insights into the molecular mechanisms of osteosarcoma (OS). However, the role of circ_0001174 in OS progression remains unknown. This study is aimed to identify differentially expressed circRNAs and messenger RNAs (mRNAs) in patients with OS and to investigate potential regulatory ways of circ_0001174.

**Methods:**

High-throughput sequencing was performed to screen aberrantly expressed circRNAs and mRNAs between tumor and paracancerous tissues from patients with OS. Several bioinformatics tools were used to analyze the functions and pathways of the differentially expressed genes between the tissues. Cell counting kit-8, cell migration and invasion assays were performed to evaluate the functions of the critical circRNAs. RNA interference experiments, quantitative real-time polymerase chain reaction (RT-qPCR) and western blotting were used to explore the relationship between miR-186-5p and circ_0001174 or metastasis-associated in colon cancer 1 (MACC1).

**Results:**

Compared with the paracancerous tissues, 109 circRNAs and 1264 mRNAs were differentially expressed in the OS tissues, including 88 circRNAs and 707 mRNAs that were upregulated and 21 circRNAs and 557 mRNAs that were downregulated. The expression of four upregulated and four downregulated circRNAs was validated using RT-qPCR; the results were consistent with the sequencing data, and circ_0001174 was found to be significantly upregulated in 16 pairs of OS tissues and OS cell lines (fold change > 2.0, *P* value < 0.05). Knockdown of circ_0001174 inhibited the proliferation, migration, and invasion of OS cells. Additionally, circ_0001174 directly and negatively modulated the expression of miR-186-5p and positively regulated the expression of MACC1.

**Conclusions:**

Abnormally high expression of circ_0001174 may promote the proliferation, migration, and invasion of OS cells through up-regulating MACC1 by sponging miR-186-5p. These results provide insight into therapeutic targets for preventing and treating OS.

**Supplementary Information:**

The online version contains supplementary material available at 10.1186/s13018-022-03059-8.

## Introduction

Osteosarcoma (OS) is a highly aggressive, metastatic bone tumor that mainly affects children and adolescents [1]. OS mainly occurs in long tubular bones such as the tibia and femur and may be accompanied by swelling of the affected limb and nocturnal pain [2]. Although surgery and adjuvant chemotherapy can effectively prolong the life of patients [3, 4], the 5‐year survival rate of OS remains at less than 20%, and the clinical prognosis is poor [5]. Therefore, a better understanding of the origin and genetic etiology of OS may lead to the development of improved strategies for the diagnosis and treatment of this tumor.

Circular RNAs (circRNAs) are a class of endogenous non-coding RNAs that form a covalently closed-loop structure and can be stably expressed in many organisms [6]. Because of their unique molecular biological characteristics, circRNAs can be used as biomarkers for disease diagnosis [7, 8]. With the development of high-throughput sequencing and experimental technology, abnormal expression of circRNAs has been widely reported in multiple cancer types, such as gastric cancer [9], cervical cancer [10], breast cancer [11], and hepatocellular carcinoma [12]. In recent years, numerous studies have indicated the important roles of circRNAs in OS, such as circECE1, circ_0002052, circ_0001721, circMYO10, among others [13–17]. However, identifying the roles of abnormal expressed circRNAs in OS is still an ongoing process. CircRNAs could function as a competitive endogenous RNA (ceRNA) which binds to microRNAs (miRNAs) to regulate gene expression [18]. Therefore, critical circRNAs, miRNAs and mRNAs, as well as their biological roles in OS tissues, should also be identified.

This study is aimed to investigate the circRNA and mRNA expression profiles of OS tissues compared with that of para-carcinoma tissues, and the roles of circ_0001174, miR-186-5p and MACC1 in OS patients, and to further confirm the underlying relationship among them.

## Methods

### Patients and specimens

Sixteen patients with primary OS (age, 10–54 years; 10 males/6 females) who underwent complete resection between May 2017 and January 2020 were recruited at the Department of Orthopedics of the Second Hospital of Jilin University. The enrolled patients were not treated with preoperative chemotherapy or radiotherapy. OS tissues and matched adjacent tissues were collected simultaneously and immediately frozen in liquid nitrogen. The Enneking staging system was used to classify the OS tumors [19]. The study was approved by the ethics committee of the Second Hospital of Jilin University (2016.169). All enrolled patients provided a signed written informed consent.

### CircRNA and mRNA sequencing

High-throughput sequencing of circRNA and mRNA in four pairs of OS tissues and adjacent tissues from patients with OS was performed at CloudSeq Biotech, Inc. (Shanghai, China). Briefly, total RNA was used to remove ribosomal RNAs using NEBNext® rRNA Depletion Kit (New England Biolabs, Ipswich, MA, USA). RNA libraries were constructed using ribosomal RNA-depleted RNAs with the TruSeq Stranded Total RNA Library Prep Kit (Illumina, San Diego, CA, USA). Libraries were controlled for quality and quantified using a BioAnalyzer 2100 system (Agilent Technologies, Santa Clara, CA, USA). Library sequencing was performed on an Illumina Novaseq 6000 instrument with 150-base pair paired-end reads. The paired-end reads were quality controlled based on Q30. After 3′ adaptor-trimming, low-quality reads were removed using the cutadapt software (v1.9.3). High-quality trimmed reads were used to analyze the circRNAs and mRNAs by aligning the reads to the reference genome/transcriptome using the STAR software (v2.5.1b), and circRNAs were detected and identified using the DCC software (v0.4.4). edgeR software (v3.16.5) was used to normalize the data and analyze differentially expressed circRNAs. The hisat2 software (v2.0.4) was used to compare high-quality reads to the human reference genome (UCSC HG19). Using the GTF gene annotation file, HTSeq software (v0.9.1) was used to obtain the original count, and edgeR was used to standardize and calculate the fold-change and P-value between the two sample sets to screen for differentially expressed mRNAs.

### Cell culture and RNA extraction

The human osteoblast cell line hFOB1.19 and OS cell lines MG63, HOS, and U2OS were acquired from the Cell Bank of Type Culture Collection of the Chinese Academy of Sciences (Shanghai, China). All cell lines were cultured in Dulbecco’s modified Eagle medium (Grand Island, NY, USA) supplemented with 10% fetal bovine serum (Grand Island, NY, USA). Total RNA was isolated using TRIzol reagent (Thermo Fisher Scientific, Waltham, MA, USA) according to the manufacturer’s instructions.

### Quantitative real-time polymerase chain reaction

Total RNA from 16 pairs of OS tissue specimens and cell lines was isolated by TRIzol (Invitrogen, USA). The RNA was reverse-transcribed into cDNA using an All-in-one First-Strand cDNA Synthesis Kit (GeneCopoeia, Rockville, MD, USA). Quantitative reverse-transcription polymerase chain reaction (RT-qPCR; ABI 7500, Applied Biosystems, Foster City, CA, USA) was performed using Power SYBR^®^ Green PCR Master Mix (Applied Biosystems). GAPDH and U6 were used as internal references. RNA expression was quantified and fold-changes in expression were determined using the 2^–△△Ct^ method. All experiments were performed in triplicate. The primer sequences used in this study are listed in Additional file 1: Table S1.

### Cell transfection

The circ_0001174 small interfering RNA (si-circ_0001174) and negative control (si-NC), overexpression (oe-circ_0001174) and negative control (oe-NC) plasmids, miR-186-5p mimic, miR-186-5p inhibitor, and negative control were synthesized by GenePharma (Shanghai, China). Lipofectamine 3000 (Thermo Fisher Scientific) was used for cell transfection according to the manufacturer's protocol. Cells were collected at 48 h after transfection for RT-qPCR analysis. Each experiment was performed in triplicate.

### Cell counting kit-8 assay

MG63 cells were seeded at a density of 3000 cells/100 µL into each well of a 96-well plate, and their proliferation ability was detected using the Cell Counting Kit-8 (Dojindo Laboratories, Kumamoto, Japan) according to the manufacturer’s protocol. The optical density was measured at 450 nm.

### Wound healing assay

Cells (1 × 10^6^) were seeded into each well of a 6-well plate and cultured for 24 h to 100% confluence. The cells were scratched with a 200-μL sterile pipette tip, washed with serum-free medium, and cultured in serum-free medium for 24 h. Images were taken immediately after wounding and at 24 h post-stimulation. The migration rate was quantified using image analysis software (ImageJ, V1.48, NIH, Bethesda, MD, USA) and calculated based on the movement of cells from the position of their initial placement to the final distance, using the following equation: (initial distance – final distance)/initial distance × 100.

### Cell migration and invasion assays

A transwell (8-mm-pore size, Millipore, Billerica, MA, USA) was used to evaluate cell migration and invasion ability. For cell migration assays, the cells (3 × 10^4^ cells in 200 μL serum-free medium) were seeded into the upper chamber, and 400 μL complete medium was added to the lower chamber. After 24 h of culture, the cells were collected. For cell invasion assays, 100 μL Matrigel (BD Biosciences, Franklin Lakes, N, USA) was first added to the chamber. The cells (3 × 10^4^ cells in 200 μL serum-free medium) were seeded into the upper chamber, and 400 μL complete medium was added to the lower chamber. After 48 h of culture, the cells were collected. The bottom of the upper membrane was fixed with 4% paraformaldehyde and stained with 0.1% crystal violet. The cells were quantified by counting five randomly selected fields under a microscope. The cells were counted using ImageJ software [20].

### Cell colony formation assay

Cells (1 × 10^3^) were seeded into each well of a 6-well plate and cultured for 10 days until colonies were clearly observed. After fixing the cells with 4% paraformaldehyde, the colonies were stained with 0.1% crystal violet solution and washed twice with phosphate-buffered saline. The colonies were counted using the ImageJ software.

### Western blotting

Total proteins were extracted from cells and tissues using 100 μL lysis buffer. The protein concentrations in the total cellular lysates were quantified using a bicinchoninic acid protein assay kit (Beyotime, Shanghai, China). Equal amounts of protein were resolved by sodium dodecyl sulfate–polyacrylamide gel electrophoresis and transferred to nitrocellulose membranes via electroblotting. After blocking with 5% skimmed milk, the membrane was incubated with the primary antibodies anti-MACC1 and anti-β-Actin at 4 °C overnight, followed by incubation with anti-rabbit horseradish peroxidase-conjugated secondary antibody (Boster, Wuhan, China). Signals were detected using an enhanced chemiluminescence detection system.

### Bioinformatics analysis

Hierarchical clustering was performed using Euclidean distance and average linkage clustering based on the circRNA and mRNA expression profiles. Gene Ontology (GO, http://www.geneontology.org/) [21, 22] and Kyoto Encyclopedia of Genes and Genomes (KEGG, http://www.genome.jp/kegg/) [23] analyses were performed to identify the roles and related pathways of differentially expressed genes between OS and paracancerous tissues. circBase (http://www.circbase.org) [24] and the Database for Annotation, Visualization, and Integrated Discovery (https://david.ncifcrf.gov/) [25] were used to annotate the differentially expressed circRNAs and mRNAs. The circRNA-miRNA interactions were predicted using circinteractome (https://circinteractome.irp.nia.nih.gov/) [26]. miRNA interaction with mRNAs was predicted using Diana tools [27], miRDB [28], and TargetScan Human 8.0 [29].

### Statistical analysis

All statistical analyses were performed using SPSS version 20.0 software (SPSS, Inc., Chicago, IL, USA). Differentially expressed circRNAs and mRNAs that significantly differed between the two groups were identified when the change in the threshold values was |fold-change|≥ 2. Comparisons between groups were performed using unpaired Student’s *t*-test. Fisher’s exact test was used to evaluate the significance of GO terms. The association between miRNA and circRNA or mRNA expression was analyzed using Spearman’s correlation coefficient. The Fisher’s exact test was performed to determine the correlation between the expression of circ_0001174 and clinical characteristics of patients with OS. Survival curves were estimated using the Kaplan–Meier method. Statistical significance was set at *P* < 0.05.

## Results

### Overview of differential expressed circRNAs and mRNAs in OS

**Fig. 1:**
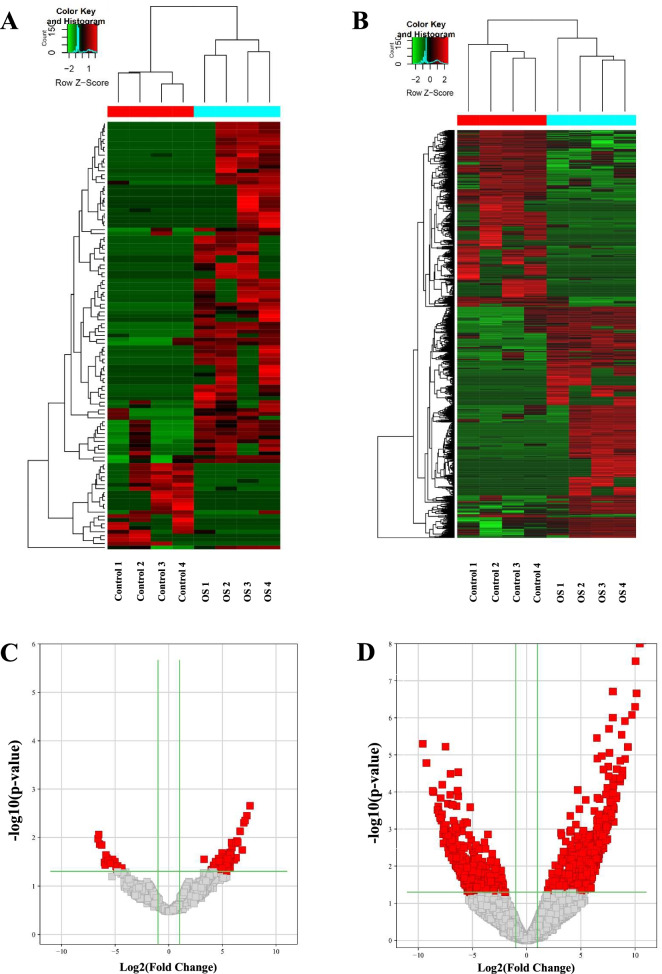
Overview of differential expressed circRNAs and mRNAs in OS. **A**, **B** Hierarchical clustering of differential expressed circRNAs (**A**) and mRNAs (**B**) in OS. Each row represents a circRNA or mRNA and each column represents an OS or control sample. Red or green colors represent high or low relative expression level, respectively. **C**, **D** Volcano plot of circRNA and mRNA expression. Each point represents a circRNA or mRNA. The red points indicate significantly differential expressed (fold-change > 2.0) circRNAs or mRNAs. RNAs were extracted from OS and matched adjacent tissues of four patients with OS

The sequencing results were evaluated using hierarchical clustering analysis, which revealed distinguishable circRNA and mRNA expression profiles between OS and adjacent tissues (Fig. 1A, B). Furthermore, up- and down-regulated circRNAs or mRNAs were displayed more intuitively in volcano plots (Fig. 1C, D). We found that 109 circRNAs and 1264 mRNAs were significantly differentially expressed, of which 88 circRNAs and 707 mRNAs were upregulated, and 21 circRNAs and 557 mRNAs were downregulated in OS tissues (Additional file 2: Table S2). The characteristics of the top 10 up- and down-regulated circRNAs are listed in Table 1.

**Table 1:** Top ten up- and down differentially expressed circRNAs in osteosarcoma

CircRNAs	Gene symbol	Regulation	logFC	Chromosome	Strand	RNA length
hsa_circ_0004001	NM_004071	Up	7.581329713	chr2	−	225
hsa_circ_0001174	TCONS_l2_00016814	Up	7.27852562	chr20	+	864
hsa_circ_0007646	NM_015115	Up	7.124993334	chr4	+	481
hsa_circ_0003423	NM_018181	Up	7.000392114	chr18	+	804
hsa_circ_0001686	NM_016447	Up	6.857675628	chr7	+	1116
hsa_circ_0001947	NM_002025	Up	6.657745525	chrX	+	861
hsa_circ_0000373	NM_178039	Up	6.450356964	chr12	+	825
hsa_circ_0102765	NM_018418	Up	6.348997278	chr14	+	922
hsa_circ_0006107	NM_002793	Up	6.279050053	chr6	−	320
hsa_circ_0120910	NM_014497	Up	6.25344204	chr2	+	955
hsa_circ_0001387	NM_007331	Down	6.610791247	chr4	+	1703
hsa_circ_0005015	NM_005328	Down	6.545988333	chr8	−	627
hsa_circ_0006848	NM_014947	Down	6.458916699	chr1	−	400
hsa_circ_0104811	T121542	Down	6.273766354	chr15	−	677
hsa_circ_0004592	NM_006281	Down	6.005818179	chr8	−	333
hsa_circ_0000348	NM_001008781	Down	5.949341164	chr11	+	3309
hsa_circ_0004826	NM_007124	Down	5.867510111	chr6	+	771
hsa_circ_0112169	NM_152495	Down	5.771157243	chr1	+	9286
hsa_circ_0024085	NM_032427	Down	5.653833409	chr11	−	1626
hsa_circ_0000448	NM_006836	Down	5.449168082	chr12	−	389

### GO and pathway enrichment analysis of differentially expressed mRNAs

**Fig. 2:**
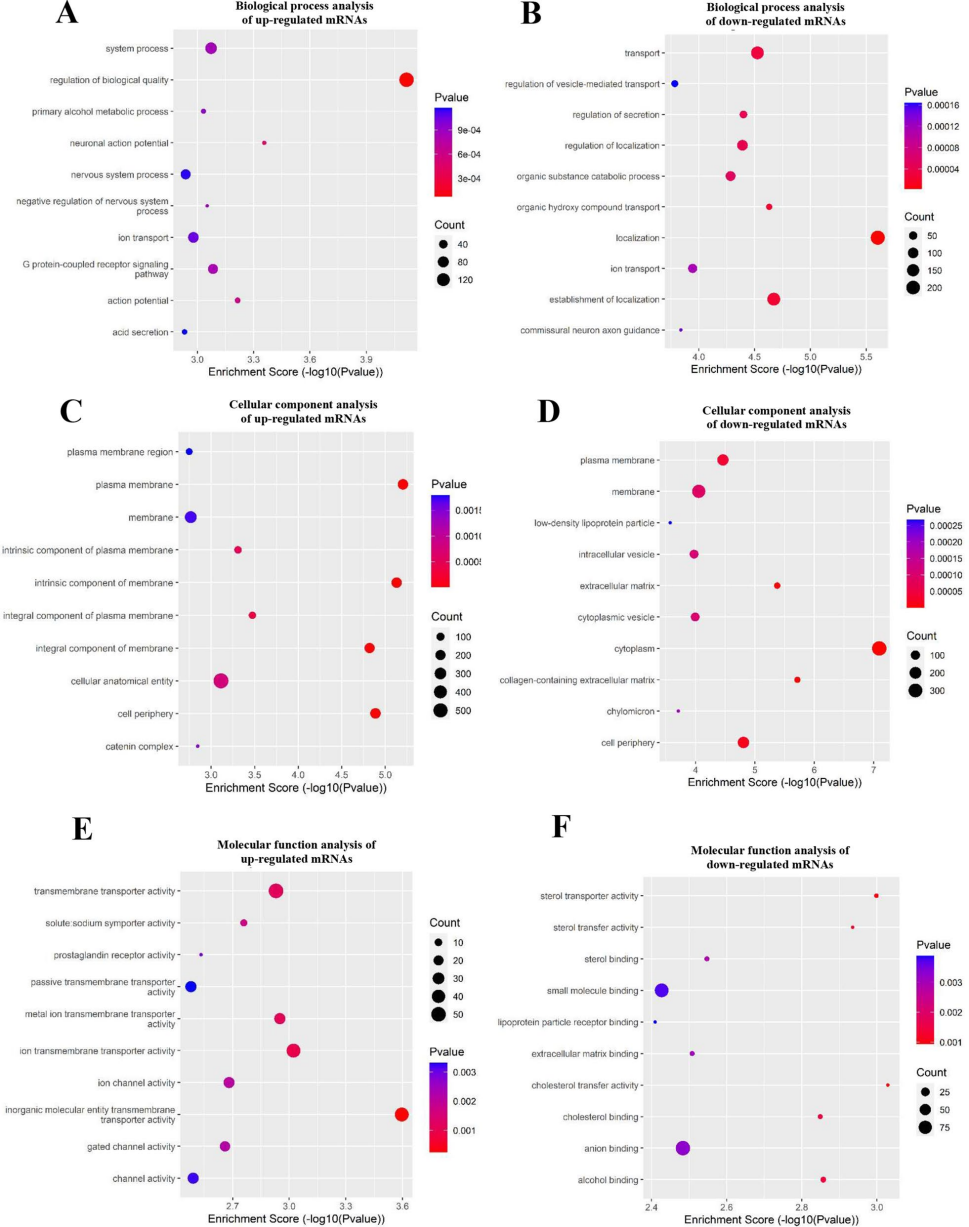

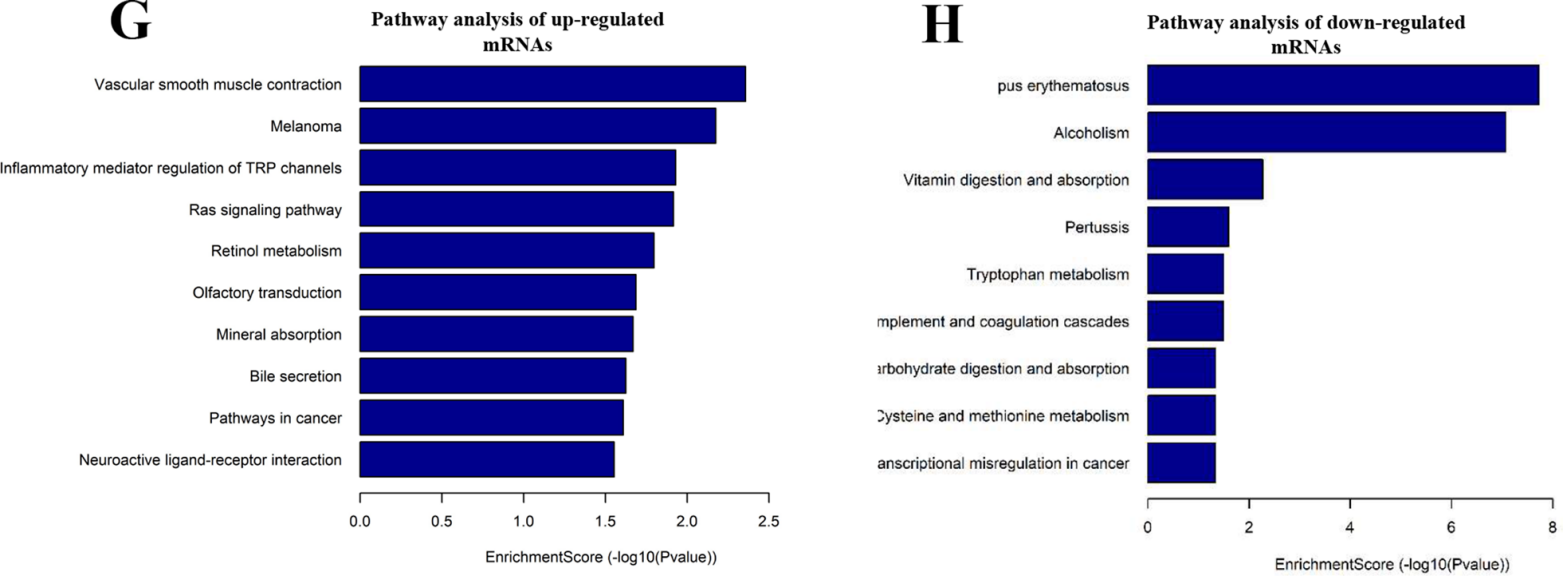
Gene ontology (GO) and pathway enrichment analysis of differentially expressed mRNAs. GO analysis covered the three domains biological process, cellular component, and molecular function. **A**–**F** Enriched upregulated and downregulated GO terms for each category. **G**, **H** Significantly upregulated and downregulated mRNA-related pathways

GO analysis was performed to determine the main functions of the differentially expressed genes. For upregulated mRNAs, regulation of biological quality, plasma membrane, and inorganic molecular entity transmembrane transporter activity showed the highest enrichment scores in biological processes, cellular components, and molecular functions, respectively (Fig. 2A, C, E). For downregulated mRNAs, localization, cytoplasm, and cholesterol transfer activity showed the highest enrichment score terms in the three categories (Fig. 2B, D, F). KEGG was used to analyze the pathways involved in abnormally expressed mRNAs. In total, 25 pathways exhibited a significant difference, including 16 pathways that included upregulated genes and nine pathways that included downregulated genes (Fig. 2G, H). All results of GO and pathway analyses are shown in Additional file 3: Table S3.

### Validation of differentially expressed circRNAs using RT-qPCR

**Fig. 3:**
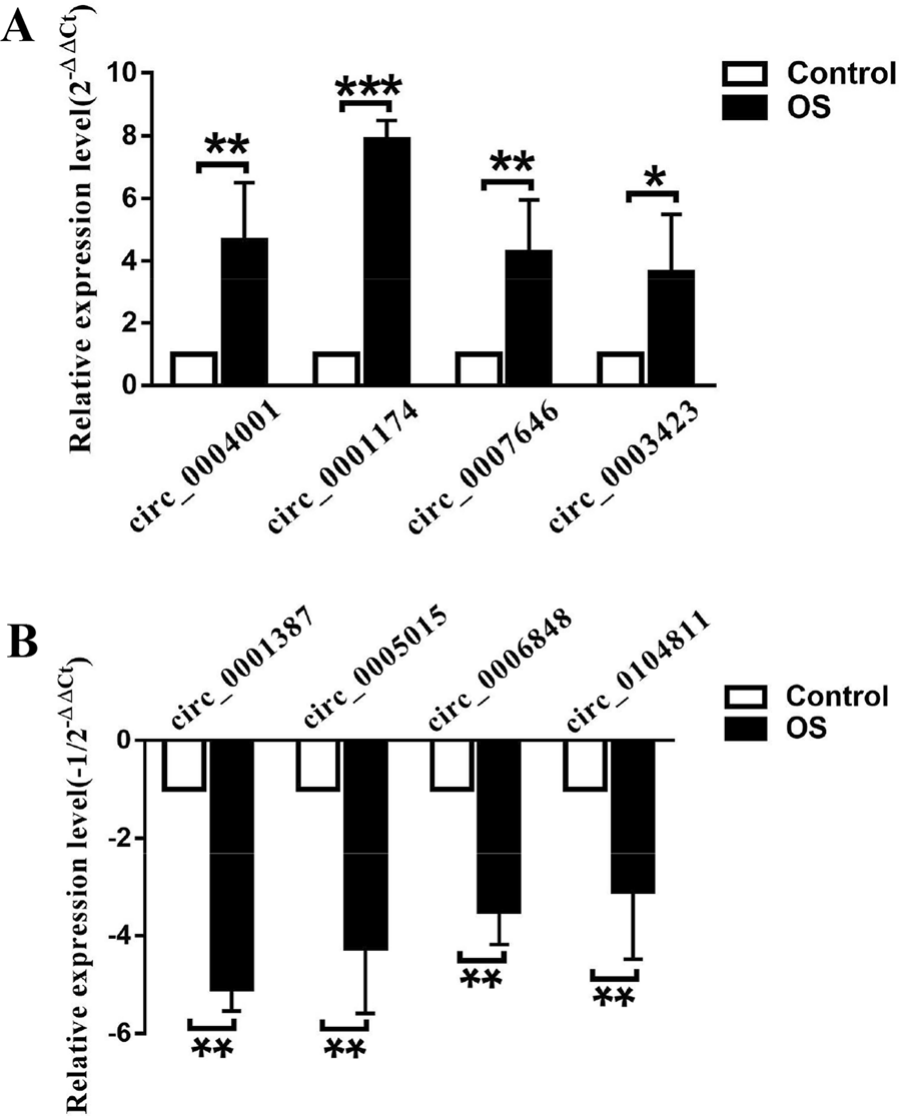
Verification of circRNA sequencing data using RT-qPCR. Top four upregulated circRNAs (**A**) and four downregulated circRNAs. **B** Sequencing data were validated using qRT-PCR of 16 osteosarcoma (OS) tissues (OS group) and 16 paired adjacent non-tumor tissues (control group). *P < 0.05, **P < 0.01, and ***P < 0.001

To confirm the reliability of the sequencing data, we selected the top four upregulated and top four downregulated circRNAs and measured their expression using RT-qPCR in 16 pairs of OS and adjacent tissues. In agreement with the sequencing data, the four upregulated circRNAs were circ_0004001, circ_0001174, circ_0007646, and circ_0003423 (Fig. 3A). The four downregulated circRNAs were circ_0001387, circ_0005015, circ_0006848, and circ_0104811 (Fig. 3B). Circ_0001174 showed the largest fold-change, and the results of the Fisher’s exact test indicated that the expression of this circRNA was closely correlated with the tumor size (Table 2). High circ_0001174 expression was closely associated with shorter disease-free survival (DFS) (Additional file 4: Figure S1).

**Table 2:** Clinical characteristics of the subjects

Parameters	Number	Circ_0001174		*P*
	(*n* = 16)	Low (*n* = 8)	High (*n* = 8)	
Age(years)	< 20	5	5	1.000
	≥ 20	3	3	
Gender	Male	5	5	1.000
	Female	3	3	
Enneking stage	IIA + IIB	7	4	0.282
	III	1	4	
Anatomic location	Femur	6	5	0.769
	Tibia	1	2	
	Humerus	1	1	
Tumor size	≤ 5 cm	6	1	*
	> 5 cm	2	7	

*P < 0.05—represent no significant statistical difference. Low expression and high expression were categorized by the median value of circ_0001174 relative expression in tumor tissues

### Circ_0001174 promotes proliferation, migration, and invasion of OS cells

**Fig. 4:**
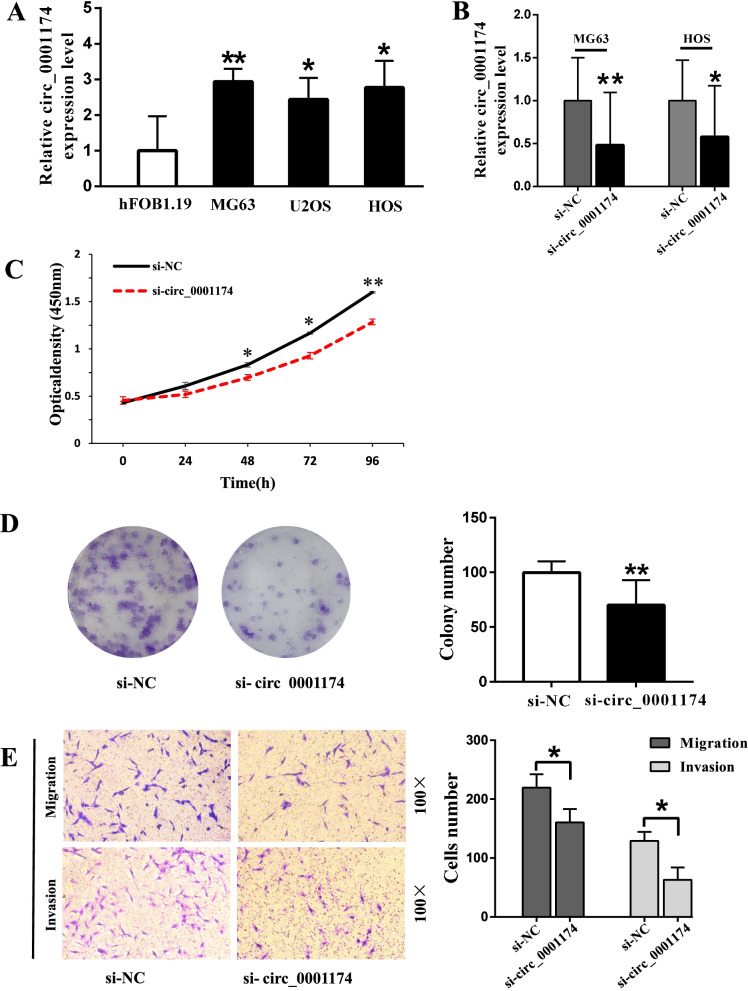

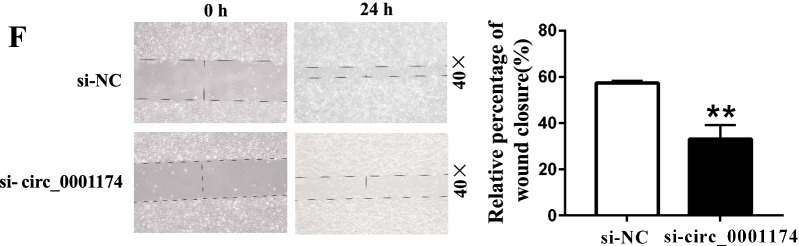
Knockdown of circ_0001174 inhibits osteosarcoma (OS) cell proliferation, migration, and invasion. **A** Expression of circ_0001174 was determined using qRT-PCR in OS cell lines and control cells (hFOB1.19 cells). **B** Circ_0001174 expression level was detected after transfection with siRNA in MG63 and HOS cells. **C** Proliferation activity of si-circ_0001174 and si-NC MG63 cells was determined using CCK-8 assay. **D** Colony formation assay was performed to analyze the proliferation ability of stably transfected MG63 cells. **E** Transwell migration and invasion assays were used to measure the migration and invasion abilities of stably transfected cells. **F** Si-circ_0001174 suppressed cell migration capacity in the wound healing assay. *P < 0.05 and **P < 0.01

As shown in Fig. 4A, the expression of circ_0001174 was significantly higher in OS cells. We transfected MG63 and HOS cells with siRNA to knock down circ_0001174 (Fig. 4B). The cell proliferation activity of MG63 cells transfected with si-circ_0001174 was significantly lower on days 2–4 (Fig. 4C). The colony-forming ability also decreased in MG63 cells transfected with si-circ_0001174 (Fig. 4D). Furthermore, the migration and invasion abilities of MG63 were prominently decreased after transfection with si-circ_0001174; these effects were confirmed by the results of the transwell and wound healing assays (Fig. 4E, F).

**Fig. 5:**
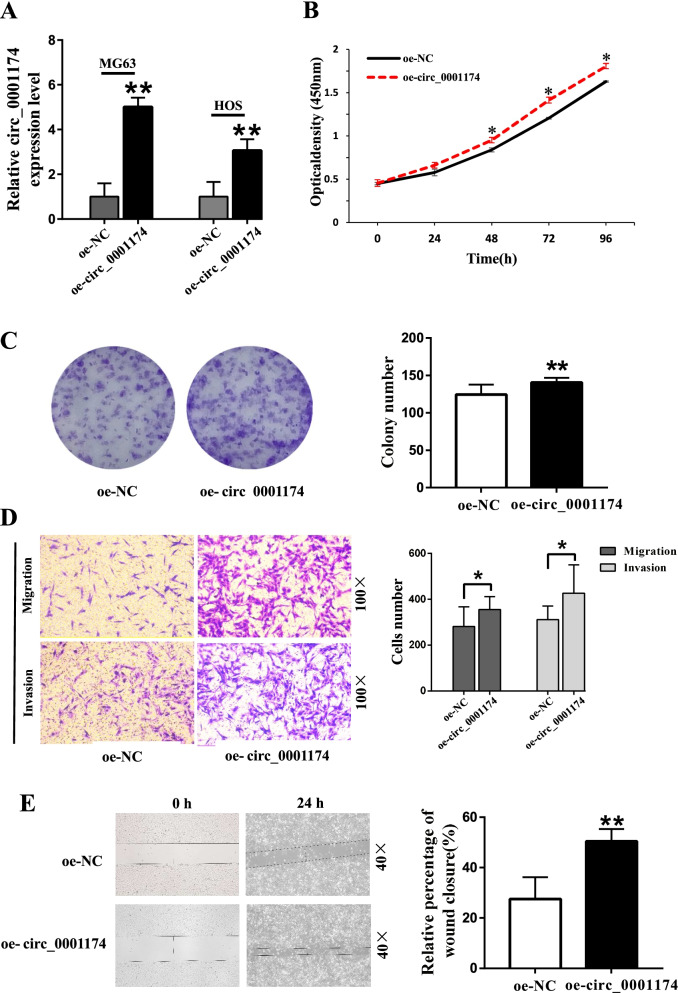
Overexpression of circ_0001174 promotes osteosarcoma (OS) cell proliferation, migration, and invasion. **A** Circ_0001174 expression level was detected after transfection with overexpression plasmids or negative control in MG63 and HOS cells. **B** Proliferation activity of oe-circ_0001174 and oe-NC MG63 cells was determined using CCK-8 assay. **C** Colony formation assay was performed to analyze the proliferation ability of stably transfected MG63 cells. **D** Transwell migration and invasion assays were used to measure the migration and invasion abilities of stably transfected cells. **E** Oe-circ_0001174 promoted cell migration capacity in the wound healing assay. *P < 0.05 and **P < 0.01

In addition, OS cell lines were transferred with the circ_0001174 overexpression plasmids or negative control (Fig. 5A). The results showed that overexpression of circ_0001174 promoted the proliferation of MG63 cells (Fig. 5B). The colony-forming ability, the migration and invasion abilities of MG63 were also increased after transfection with oe-circ_0001174, respectively (Fig. 5C, D, E). These effects were confirmed by the results of the transwell and wound healing assays (Fig. 5F).

### Circ_0001174 is a sponge for miR-186-5p

**Fig. 6:**
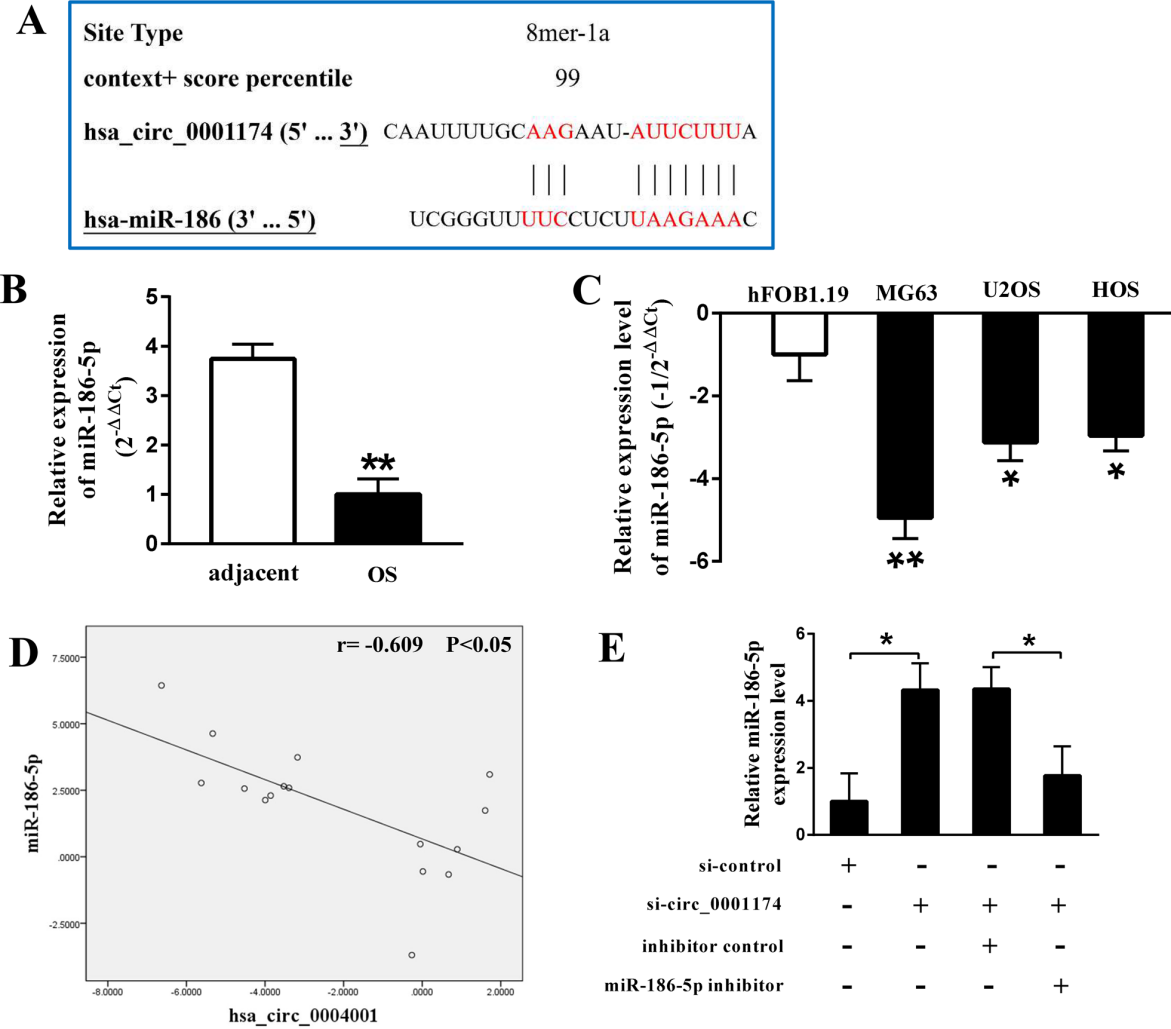
Circ_0001174 serves as a sponge for miR-186-5p. **A** Bioinformatics analysis showed that miR-186-5p is a potential target of circ_0001174. **B**, **C** Expression of miR-186-5p was significantly downregulated in OS tissues and cell lines. **D** Circ_0001174 expression was negatively correlated with miR-186-5p expression in OS tissues. **E** After MG63 cells were transfected with si-circ_0001174, miR-186-5p expression was significantly upregulated, which was reversed by miR-186-5p inhibitor

Based on the competing endogenous RNA mechanism in which circRNAs can compete for binding to microRNAs to regulate mRNA expression [30], we predicted the potential targets of circ_0001174 using circinteractome. As shown in Fig. 6A, there were clear binding sites between circ_0001174 and miR-186-5p (context + score percentile = 99). In addition, miR-186 expression levels were significantly downregulated in OS tissues compared to adjacent normal tissues (16 pairs of samples, Fig. 6B). The same results were found in the OS cell line, particularly in MG63 cells compared to in hFOB 1.19 cells (Fig. 6C). In addition, correlation analysis was performed to investigate the relationship between circ_0001174 and miR-186-5p expression. As shown in Fig. 6D, circ_0001174 expression was negatively correlated with miR-186-5p expression. Furthermore, miR-186-5p expression was significantly upregulated after MG63 cells were transfected with si-circ_0001174, which was reversed by the miR-186-5p inhibitor (Fig. 6E).

### MACC1 is a direct target of miR-186-5p in OS

**Fig. 7:**
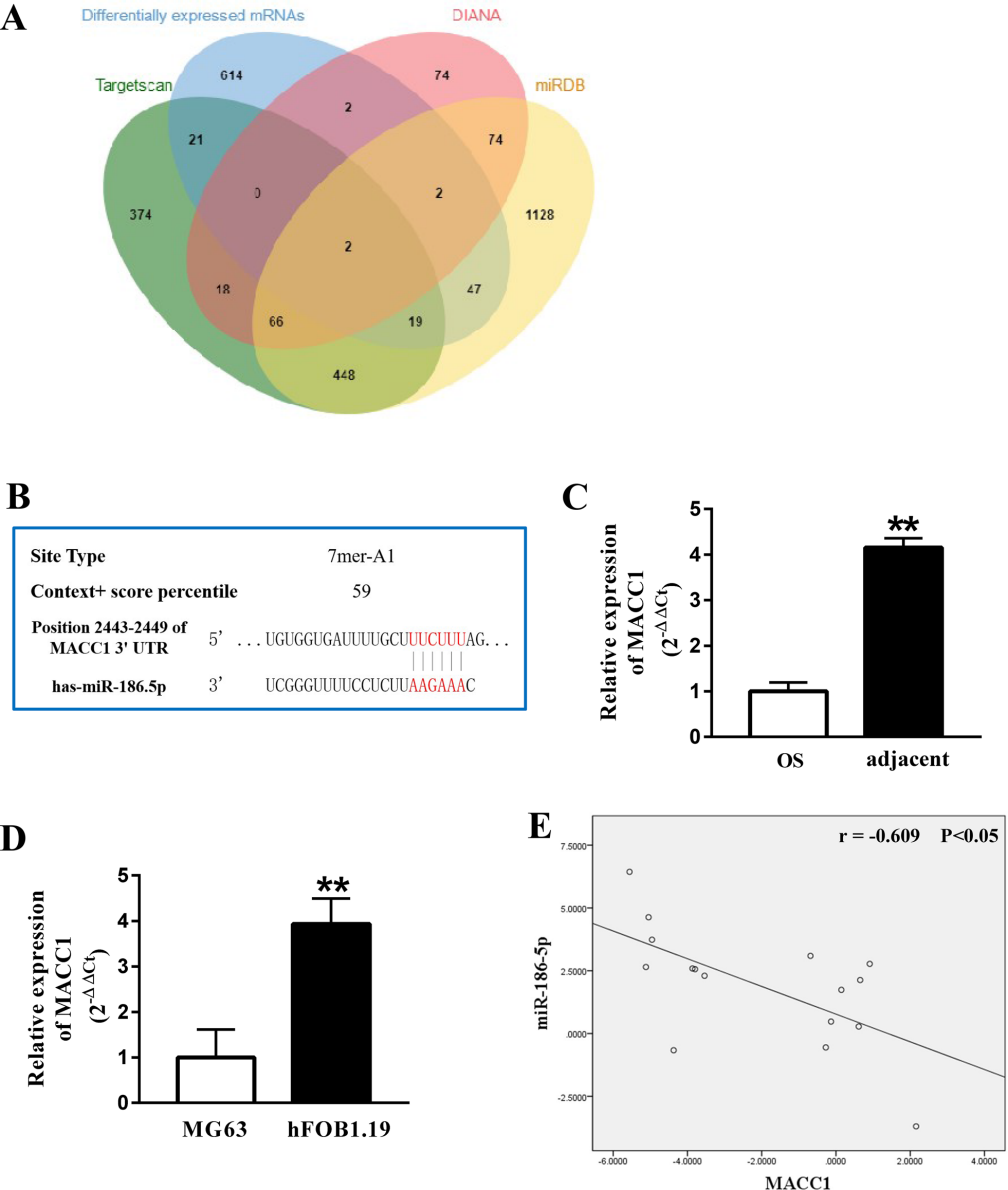

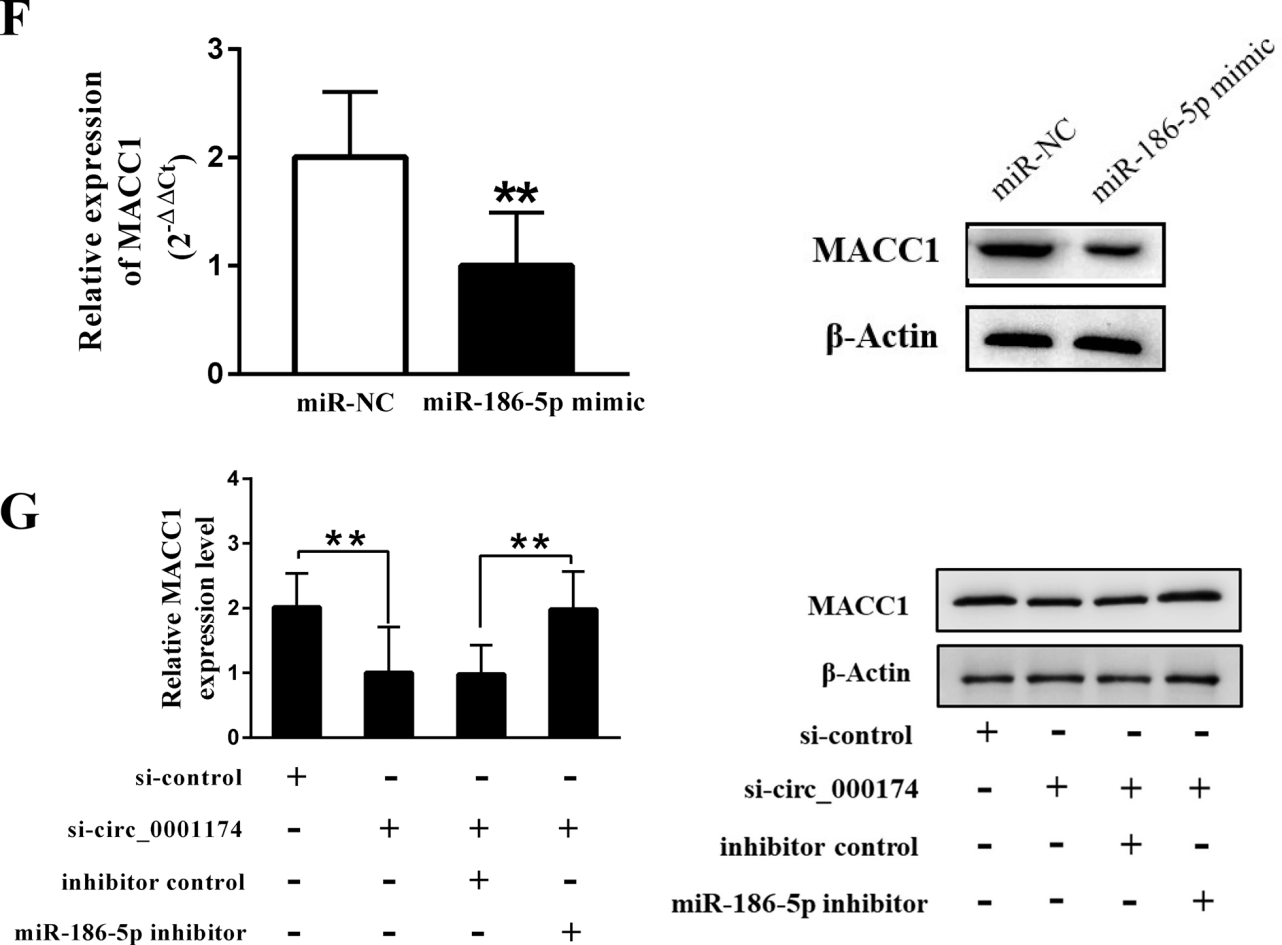
Circ_0001174 could regulate MACC1 expression by sponging miR-186-5p. **A** The Venn diagram was contained 3 databases (Targetscan, DIANA tools and miRDB) predicted binding mRNAs and differentially expressed mRNAs. **B** The binding site between miR-186-5p and MACC1. **C**, **D** The expression of MACC1was significantly increased in OS tissues (16 pairs of OS and adjacent tissue) and MG63 cells. **E** The negative correlation was found between miR-186-5p and MACC1. **F** After transfected with miR-186-5p mimic, the expression of MACC1 was detected by qRT-PCR and western blot, respectively. **G** qRT-PCR and western blot analysis showed miR-186-5p inhibition could rescue suppressed effect caused by si-circ_0001174 in the expression of MACC1. Results represented the mean ± s.d. of three independent experiments and the relative expression levels of each gene were analyzed using the 2^−△△Ct^ method. ** represent *P* < 0.01

We predicted the potential targets of miR-186-5p using TargetScan, DIANA tools, and miRDB. As shown in the Venn diagram (Fig. 7A), the intersection showed two differentially expressed mRNAs: MACC1 (metastasis-associated in colon cancer-1) and LHFPL2 (lipoma HMGIC fusion partner-like 2). Based on database prediction of the binding ability score and ranking order of the sequencing results, we further analyzed MACC1. The binding sites between miR-186-5p and MACC1 are shown in Fig. 7B. Furthermore, the expression of MACC1was significantly increased in OS and MG63 cells (Fig. 7C, D). Spearman’s rank correlation analysis revealed a negative correlation between miR-186-5p and MACC1 in OS tissues (Fig. 7E). In addition, overexpression of miR-186-5p inhibited MACC1 expression at both the mRNA and protein levels (Fig. 7F, G).

## Discussion

OS is the most common primary malignant bone tumor in orthopedics, and often affects children and young adults [31]. This highly aggressive bone tumor typically has a poor prognosis. Although current treatments involve surgical resection, radiotherapy, and combinations of double or triple chemotherapy, the recurrence rate within 5 years is approximately 30% [32–34]. This seriously affects the quality of life of patients and creates challenges for orthopedic surgeons. Therefore, a detailed understanding of OS from the genetic and molecular perspectives is important for improving the treatment and quality of life of patients.

An increasing number of studies have shown that circRNAs can serve as competing endogenous RNAs to regulate mRNA expression by reducing the levels of miRNAs. Ji et al. found that circ_001621 promoted OS cell proliferation and migration by sponging miR-578 and regulating vascular endothelial growth factor [35]. Ma et al. confirmed that circ_0007142 sponges miR-186 and regulates FOXK1 expression, leading to the progression of lung adenocarcinoma [30]. Lu et al. reported that circRNA_100876 suppresses the proliferation of OS cancer cells by targeting microRNA-136 [14]. However, the function of circ_0001174 remains unknown.

We determined the circRNA and mRNA expression profiles of OS tissues compared to those of adjacent tissues. A total of 109 circRNAs and 1264 mRNAs were differentially expressed (fold-change ≥ 2, *P* value < 0.05). Among these, we focused on the role and underlying mechanisms of circ_0001174 in OS progression. We confirmed that circ_0001174 expression was abnormally increased in both OS tissues and cells. Furthermore, knockdown of circ_0001174 inhibited OS cell proliferation, migration, and invasion. The regulatory mechanisms involved in this abnormal presentation were further examined. Bioinformatics analysis and experimental validation suggested that miR-186-5p is a target of circ_0001174. Specifically, the expression of miR-186-5p and circ_0001174 was negatively correlated, and circ_001621 expression was significantly decreased in both OS tissues and cells. Several other studies have revealed that miR-186-5p is involved in the abnormal regulation of OS as a tumor suppressor [36–38]. In addition, the long non-coding RNAs NEAT1 and DSCAM-AS1 have been reported to sponge miR-186-5p to regulate downstream genes in OS [39, 40]. We revealed a competitive inhibitory relationship between circ_0001174 and miR-186-5p, and showed that the abnormally high expression of circ_0001174 is involved in the development of OS.

We predicted the downstream gene of the circ_0001174/miR-186-5p axis using three databases. MACC1 was significantly increased in both OS tissues and cells and was confirmed to be a target of miR-186-5p. MACC1 is localized on human chromosome 7, which contains seven exons and six introns [41]. Previous studies reported that high MACC1 expression predicts poor prognosis in patients with OS and is closely correlated with the clinical stage and distant metastasis [42]. Additionally, several studies have described the pathways regulated by MACC1 in OS, such as the HGF/c-Met and Akt signaling pathways [43, 44]. MACC1 was also confirmed to be a direct target of miRNA-432 for inhibiting cell proliferation and invasion in OS [45]. Similar to the results of previous research, we found that MACC1 levels were abnormally high in OS tissues and cells. Furthermore, the miRNA-186-5p mimic significantly reduced the expression of MACC1 mRNA and protein. Thus, we revealed the regulatory relationship between miR-186-5p and MACC1.

In recent years, non-coding RNAs have been found to play important roles in the development of orthopedic diseases [46–48]. When mRNAs cannot fully explain the pathogenic mechanism of a disease, the involvement of circRNA, a type of non-coding RNA, in the mechanism should be evaluated. Although we evaluated circ_0001174 and obtained several significant results, there are many other potentially meaningful circRNAs and mRNAs that should be examined in detail.

There were some limitations to our study. First, our OS tissue sample size for sequencing analysis was small, which may have affected the circRNA and mRNA expression profile results. Second, most abnormally expressed circRNAs and their exact targets must be validated. Finally, luciferase reporter assays and animal experiments should be performed.

## Conclusions

We identified 109 circRNAs and 1264 mRNAs that were abnormally expressed in OS tissue samples. Furthermore, circ_0001174 was found to promote OS cell proliferation and invasion by targeting the miR-186-5p/MACC1 axis. Our results improve the understanding of the molecular mechanisms underlying OS and provide therapeutic insights for patients with OS.

## Supplementary Information

**Additional file 1.** Primers for circRNAs mRNAs and miRNAs in real-time RT-PCR.

**Additional file 2.** Differential expressed circRNAs and mRNAs in OS tissues.

**Additional file 3.** GO and pathway analyses results.

**Additional file 4.** Correlation of circ_0001174 expression with DFS.

## Data Availability

The data used to support the findings of this study are available from the corresponding author upon request.
