# Supplementary material for: Circ_0001174 facilitates osteosarcoma cell proliferation, migration, and invasion by targeting the miR-186-5p/MACC1 axis
Source: J Orthop Surg Res. 2022 Mar 12;17:159. doi: 10.1186/s13018-022-03059-8 (PMC8917736; doi:10.1186/s13018-022-03059-8)
Supplement: Supplementary file 1 — Additional file 1. Primers for circRNAs mRNAs and miRNAs in real-time RT-PCR. [file 13018_2022_3059_MOESM1_ESM.doc]

**Supplemental Table 1**. Primers for circRNAs mRNAs and miRNAs in real-time RT-PCR

| **Gene** | **Primer sequences** | |
| --- | --- | --- |
| circ_0004001 | F: 5’- ACGAACATCACAGTACATTGGT -3’ |  |
|  | R: 5’- TTGGGATTATACGCCTCTGTG -3’ |  |
| circ_0001174 | F: 5’- CTTGTGAAGGCGGTGCTG -3’ |  |
|  | R: 5’- GAAGCCCTTGAATGAAGCCC -3’ |  |
| circ_0007646 | F: 5’- AGCTTGGAAATTGGATGCACA -3’ |  |
|  | R: 5’- TGTTTGGTGATCGTCTTGGC -3’ |  |
| circ_0003423 | F: 5’- GTGTGACTGCCTGTTCATGC -3’ |  |
|  | R: 5’- GGGCAGGTGTAGGGAGATTT -3’ |  |
| circ_0001387 | F: 5’- GGACACACCCAGGAAAAGAC -3’ |  |
|  | R: 5’- GCTAAATTCCATCCAGCCCAG -3’ |  |
| circ_0005015 | F: 5’- GCAACACGTAACGCAATTGG -3’ |  |
|  | R: 5’- GCAGCTGTGATTCCAAGGAG -3’ |  |
| circ_0006848 | F: 5’- TATAGAGAGGCTGGCAGTGG -3’ |  |
|  | R: 5’- TGGGTCAAGGAGTGCATTCT -3’ |  |
| circ_0104811 | F: 5’- CTGAGAGAGGCTGGAAGGAA -3’ |  |
|  | R: 5’- CTGGAGATTCACCCACCTGT -3’ |  |
| miR-186-5p | F: 5'- AAGAATTCTCCTTTTGGGCT -3' |  |
|  | R: 5'- GTGCGTGTCGTGGAGTCG -3' |  |
| miR-186-5p-mimics | 5'- CAAAGAAUUCUCCUUUUGGGCU-3' |  |
| miR-186-5p- inhibitor | 5'- AGCCCAAAAGGAGAAUUCUUUG -3' |  |
| MACC1 | F:5’-CATTTTCGGTCAGGAAGAATTGCT-3’ |  |
|  | R:5’- TGGAAGCATTATTACCACGAAGG -3’ |  |
| GAPDH | F: 5’- CGGACCAATACGACCAAATCCG -3’ |  |
|  | R: 5’- AGCCACATCGCTCAGACACC -3’ |  |
| U6 | F: -GCTTCGGCAGCACATATACTAAAAT-3’ |  |
|  | R: -CGCTTCACGAATTTGCGTGTCAT-3’ |  |

GAPDH, glyceraldehyde-3-phosphate dehydrogenase. MACC1, metastasis associated in colon cancer 1.
