# Supplementary material for: Circ_0001174 facilitates osteosarcoma cell proliferation, migration, and invasion by targeting the miR-186-5p/MACC1 axis
Source: J Orthop Surg Res. 2022 Mar 12;17:159. doi: 10.1186/s13018-022-03059-8 (PMC8917736; doi:10.1186/s13018-022-03059-8)
Supplement: Supplementary file 4 — Additional file 4. Correlation of circ_0001174 expression with DFS. [file 13018_2022_3059_MOESM4_ESM.docx]

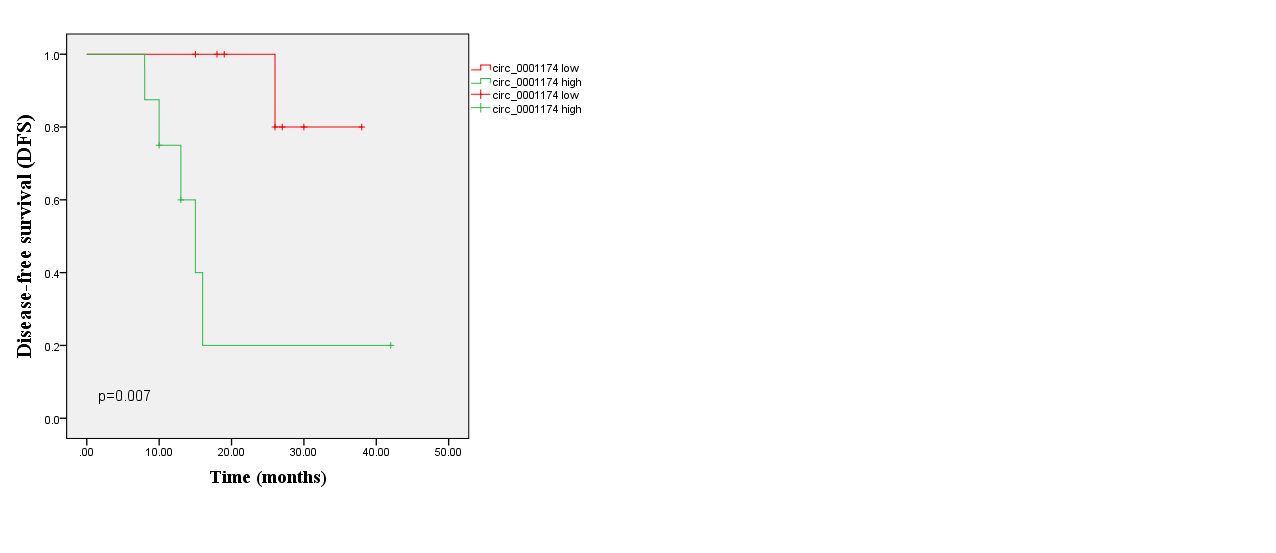

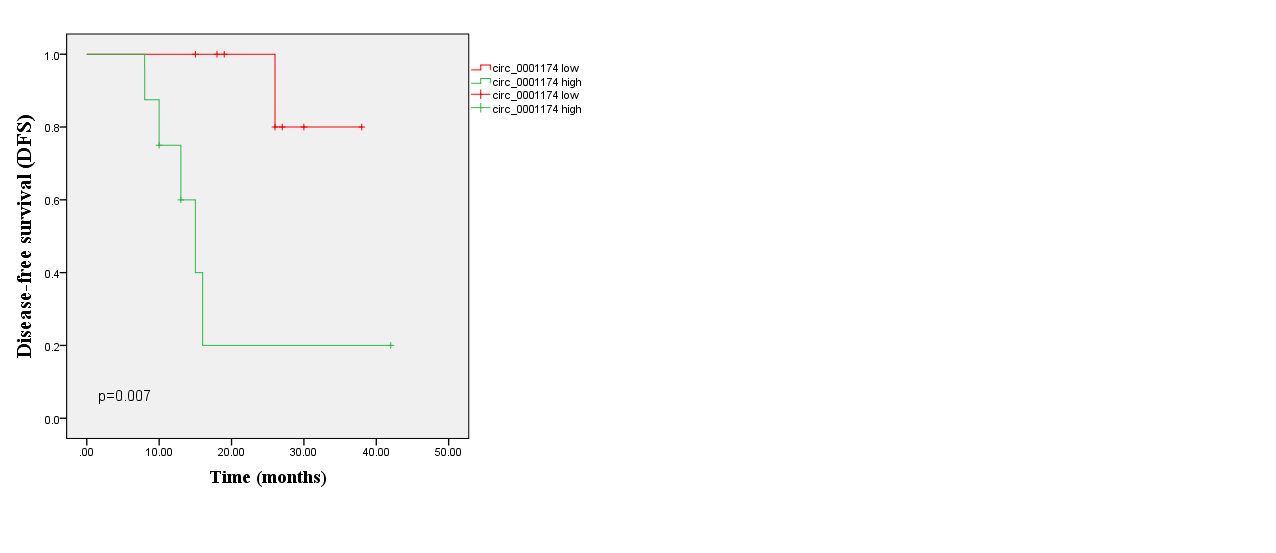


**Supplementary figure. Correlation of circ_0001174 expression with DFS.**

The correlation of circ_0001174 expression with DFS. Based on the follow‐up data, DFS was calculated for survival analysis. High circ_0001174 expression was closely associated with shorter DFS. P value <0.05 was considered statistically significant.
